# Temporal patterns of adolescent screen time and compulsive internet use in Moroccan high school students

**DOI:** 10.1016/j.abrep.2026.100667

**Published:** 2026-01-13

**Authors:** Samiha Imrani, Bouzekri Touri, Lucia Romo, Oulmann Zerhouni

**Affiliations:** a Laboratory of Information and Education Sciences and Technologies (LASTIE), Faculty of Sciences Ben M’Sik, Hassan II University of Casablanca, Casablanca, Morocco; b University Paris Nanterre UR 4430 Departement of Psychology, 200 av de la république, 92001 Nanterre Cedex, France; c Hôpital Universitaire Raymond-Poincaré, Service de Pathologies Professionnelles (AP-HP), 92380 Garches, France; d Inserm CESP 1018 UPS, 114, rue Edouard-Vaillant, 94 805 Villejuif Cedex, France; e Univ. Rouen Normandie, CRFDP, Mont-Saint-Aignan, France; f Univ. Paris Nanterre, LAPPS, Nanterre, France; g Institut pour le Développement et l’Action en Sciences Sociales (IDASC), France

**Keywords:** Adolescents, Screen time, Compulsive internet use, Morocco, Latent class analysis, Growth modeling

## Abstract

**Objective**: Although compulsive internet use reflects interactions between personal vulnerabilities and situational triggers, adolescents’ day-to-day screen time patterns, especially in non-Western contexts, remain understudied. We aim to examine temporal patterns of screen time among Moroccan adolescents and their associations with compulsive internet use (CIU) and sociodemographic factors. **Methods**: This study uses an intensive daily design to capture short-term fluctuations in screen use across a week, identifying weekday–weekend shifts and other dynamics. A total of 334 Moroccan high school students (M_age_ = 16.02 years, 52 % female) from three public schools in urban and rural areas reported their daily screen time and CIU over seven consecutive days, their platform-specific usage and their scores on the CIUS. Latent class analysis (LCA) was used to identify subgroups with distinct weekly trajectories, and latent growth modeling (LGM) was used to assess temporal change. Linear mixed-effects models tested predictors, including age, sex, CIUS scores, school type, device preference, and primary internet activity. **Results**: Two distinct usage profiles emerged: steady-low and weekend-high. The weekend-high profile was more common among older girls and showed strong Sunday peaks. Mixed-effects and latent class models showed that higher CIUS scores, being female, and the day of the week (especially Sunday, with an increase of 74 min compared to weekdays) predicted heavier, multi-platform engagement. **Conclusions**: Moroccan adolescents show heterogeneous temporal patterns of screen use, with compulsive use linked to heavier, weekend-centered engagement. Prevention efforts should target overall exposure and high-risk time windows, especially on weekends.

## Highlights

• First LCA of adolescent screen time patterns in a Moroccan context.
• Weekend peaks strongly linked to compulsive internet use scores.
• Combined LCA and latent growth modeling to capture temporal variability.
• Boys spent more time gaming; girls favored social media platforms.
• Findings inform culturally tailored prevention and policy strategies.

## 1 Introduction

Adolescence is a critical developmental stage marked by rapid physical, cognitive, and socioemotional changes (Steinberg, 2014) that are increasingly shaped by pervasive smartphone and internet connectivity. While digital technologies offer educational and social benefits, concerns have been raised about the effects of excessive smartphone use on mental health, academic performance, and overall well-being (Kuss and Griffiths, 2017, Soriano-Molina et al., 2025). Over the past decade, digital screens—including smartphones, tablets, computers, and gaming consoles—have become embedded in adolescents’ daily routines. CIU, sometimes referred to as internet addiction, is especially prevalent during adolescence due to the increased tendency toward compulsive behaviors and impulsivity (Anderson et al., 2017). CIU refers to patterns of online engagement that interfere with daily functioning and are associated with emotional, behavioral, or cognitive difficulties (Meerkerk et al., 2009). Within this broader category, CIU reflects core symptoms of dysregulation, including preoccupation, loss of control, and repeated unsuccessful attempts to limit use. This aligns with models of addictive or compulsive behavior (Brand et al., 2016, Meerkerk et al., 2009). These features make compulsive use a key risk factor for heavier and more disruptive digital engagement, particularly among adolescents whose developing self-regulatory capacities make them vulnerable to such behaviors (Steinberg, 2014). In the present study, CIU is examined as a theoretically grounded predictor of weekly screen time patterns.

First, we outline why adolescence is a critical period for understanding digital engagement. Then, we define CIU and its correlates. Next, we present the theoretical framework guiding the study and explain the methodological relevance of a daily intensive design in the Moroccan context. Finally, we identify the research gap that motivates our objectives and hypotheses.

### 1.1 Global prevalence and associated risks

Global surveys highlight the prevalence of digital engagement among adolescents. In the United States, for instance, 95 % of adolescents own a smartphone, and 89 % go online daily, averaging over four hours of screen time on weekdays (Lenhart et al., 2015). Similar patterns have been observed in Europe, where older adolescents tend to engage more heavily than their younger peers (Rideout, 2016). Excessive use has been linked to poor sleep, lower academic performance, and emotional difficulties. Longitudinal evidence shows that nighttime device use predicts shorter, lower-quality sleep (Hysing et al., 2015, Przybylski and Weinstein, 2017). This, in turn, is associated with antisocial behaviors (Bègue et al., 2022) and cognitive impairments (Imrani and Touri, 2025a, Baba et al., 2024). Greater social media and smartphone use is also associated with higher rates of depression and anxiety among adolescents (Keles et al., 2020).

Adolescence is indeed a period of significant change, creating a unique context for digital engagement. According to neurodevelopmental models, adolescents exhibit heightened responsivity to reward and socioemotional systems, as well as protracted maturation of cognitive control. This increases their sensitivity to salient, socially rewarding stimuli and their susceptibility to compulsive or dysregulated behaviors (Casey et al., 2008, Crone and Dahl, 2012, Spear, 2000). Epidemiological data indicate that many mental health and substance use disorders first emerge during adolescence and early adulthood. This underscores the critical nature of this period for the onset of problematic behaviors (Kessler et al., 2005). Consistent with this, meta-analytic studies indicate that problematic internet and social media use are prevalent among adolescents and are associated with increased depression, anxiety, and general psychological distress in this age group (Keles et al., 2020, Shannon et al., 2022). Taken together, these findings justify a focus on adolescents when examining temporal patterns of screen use and their association with CIU. Problematic internet use (PIU) and problematic social media use (PSMU) are often used as umbrella terms in the literature; in the present study, we focus specifically on compulsive internet use (CIU), which captures the core dysregulation component of excessive online behavior.

### 1.2 Theoretical framework: I-PACE and behavioral correlates

In the present study, compulsive internet use (CIU) is conceptualized as a persistent difficulty in regulating internet-related behaviors, characterized by preoccupation with going online, loss of control, and continued use despite negative consequences. This conceptualization follows the theoretical foundations of the Compulsive Internet Use Scale (CIUS) and aligns with established models of dysregulated and compulsive internet use rather than internet use per se (Meerkerk et al., 2009, Brand et al., 2016). Within this framework, CIU represents a core behavioral marker of dysregulation that is directly observable through patterns of digital engagement over time. Empirical research has shown that higher CIU scores are associated with longer and less controllable online sessions, including when compared with objective indicators of device use (Boase and Ling, 2013, Van Deursen et al., 2015). Importantly, compulsive use and total screen time are only moderately correlated, indicating that high exposure does not necessarily imply dysregulation and that CIU captures a distinct dimension of problematic engagement (Ryding & Kuss, 2020). Accordingly, the present study treats CIU as a theoretically grounded predictor of short-term fluctuations in adolescents’ daily screen time.

Empirical studies consistently show that compulsive internet use is positively associated with total screen time, but that the magnitude of this association is moderate rather than redundant. For example, Meerkerk et al. (2009) reported that higher CIUS scores were related to longer online sessions, yet substantial variability in screen time remained unexplained by compulsive use alone. Similarly, device-logged studies indicate that individuals with elevated compulsive-use scores tend to engage in longer and less controllable digital sessions, but that high screen time can also reflect goal-directed or instrumental activities that are not inherently compulsive (Van Deursen et al., 2015, Ryding and Kuss, 2020). These findings suggest that screen time and compulsive internet use capture related but non-identical aspects of digital engagement, supporting the need to examine both constructs simultaneously when investigating adolescents’ daily usage patterns.

A substantial body of research also indicates systematic sex differences in compulsive internet use during adolescence. Girls tend to report higher levels of compulsive or dysregulated online engagement than boys, particularly on social and communication platforms (Keles et al., 2020, Van Deursen et al., 2015). These differences are directly relevant to the present study, which examines how CIU and sociodemographic factors, including sex, relate to short-term variations in daily screen time.

### 1.3 Daily fluctuations, methodological rationale, and cultural context

Although research on problematic smartphone use has grown substantially, most studies rely on aggregated daily or weekly averages, overlooking temporal variability within the week. A few studies that have examined daily fluctuations suggest that adolescents' screen time often peaks on weekends, possibly due to differences in school schedules, social opportunities, and parental supervision (Hysing et al., 2015, Rideout, 2016). However, these findings predominantly stem from Western contexts and may not generalize to countries with distinct cultural rhythms. In the Moroccan context, the daily routines of adolescents differ from those commonly reported in Western settings. School days tend to begin earlier and end later. Extended family households are more common, and weekend leisure activities tend to be communal and family-based rather than individual extracurricular pursuits (Bouazza et al., 2023). These cultural rhythms may shape unique patterns of digital engagement throughout the week. We examine these patterns in a non-Western context to address this critical gap in the literature and provide a culturally grounded perspective on smartphone usage dynamics.

In the present study, we use the term “temporal pattern” to describe short-term, within-week fluctuations in adolescents’ digital engagement. These fluctuations arise from situational triggers—including school schedules, social opportunities, and family routines—that vary markedly across the days of a week. Because these dynamics unfold over very short time intervals, they cannot be adequately captured through prospective longitudinal designs that focus on changes across months or years. Instead, we adopted a seven-day intensive daily design to detect meaningful within-week patterns, such as weekday–weekend shifts, that are central to theoretical models of dysregulated and CIU.

### 1.4 Research gap in North Africa and hypotheses

Despite the rapid growth of research on adolescents’ digital engagement, most studies rely on cross-sectional or prospective designs that use aggregated daily or weekly averages of screen time. While these approaches provide insight into overall exposure, they are not well-suited for capturing short-term, within-week fluctuations that may reflect situational triggers, regulatory demands, and daily routines. Very few studies have collected day-to-day estimates of adolescents’ screen use over consecutive days, and even fewer have combined such intensive measurement with person-centered methods and growth modeling. Consequently, we still know little about the relationship between CIU and sociodemographic factors, such as age and sex, and concrete daily usage patterns across a typical week, especially in non-Western settings, like Morocco (Bouazza et al., 2023).

Short-term fluctuations are difficult to capture with aggregated weekly averages or long-term prospective designs because they unfold over short intervals. This motivates the use of person-centered and time-sensitive statistical approaches. Latent class analysis identifies subgroups of adolescents with different within-week usage trajectories, while nonlinear growth models show how screen time changes across days. Mixed-effects models estimate how individual and contextual factors shape daily use. This study aims to address this gap by integrating a seven-day LCA of self-reported daily screen time with LGM. We modeled daily trajectories using the Richards growth curve, a flexible sigmoidal function that allows for asymmetry and variable inflection points (Bates & Watts, 1988). We chose this approach over polynomial or spline approaches because it can capture realistic plateau–peak–plateau patterns without overfitting (Ritz & Streibig, 2009). We examined whether higher CIUS scores and objective app usage predict usage trajectories and whether age, sex, and school context moderate these patterns. Our hypotheses are as follows: (a) distinct classes will emerge (e.g., “weekend peakers” or “steady high users”), (b) CIUS and heavier app usage will predict membership in higher-use or steeper-growth profiles, and (c) demographic predictors will exert weaker effects.

## 2 Method

### 2.1 Participants

The sample comprised 334 high school students recruited from three institutions in the Casablanca region of Morocco. The participants were between 14 and 18 years old (Mage = 16.02 years, SD = 1; 52 % female). There were no exclusion criteria regarding disability, and the study did not collect diagnostic information about neurodevelopmental or physical disabilities. Sex was recorded as a binary variable (male/female) based on self-report and school records; no measure of gender identity was collected. Males and females had similar ages (both groups M = 16.0 years, SD = 1.0), indicating comparable age distributions across sex. One school was situated in a rural area and two in urban settings to capture potential contextual differences in Internet use. Participation was voluntary and contingent upon written informed consent from both students and their legal guardians.

### 2.2 Ethical considerations and informed consent

This study was conducted in accordance with the ethical principles outlined in the Helsinki Declaration of 1975 (revised in 2000). As no institutional ethics committee was available at the university, the research was officially authorized by the Regional Academy of Education under reference number 54/10/24 and approved by the Dean of the Faculty of Letters and Human Sciences Ben M’sik, Hassan II University of Casablanca. Since the participants were minors, informed consent was obtained from parents or legal guardians, and assent was secured from all adolescent participants prior to data collection. Participants were informed about the study’s objectives, the confidentiality of their responses, and their right to withdraw at any time. No identifying information is included in this article. All collected data were anonymized to ensure participant privacy and ethical compliance.

## 3 Measures

Compulsive Internet Use. CIU was assessed using the 14-item CIUS (Meerkerk et al., 2009), which measures five core dimensions of dysregulated online behavior: (a) Loss of control (e.g., ‘I find it difficult to stop using the internet when I am online’), (b) Preoccupation (e.g., ‘I think about the internet even when I am not online’), (c) Withdrawal symptoms (e.g., ‘I feel restless when I cannot use the internet’), (d) Coping/mood regulation (e.g., ‘I use the internet to escape from my sorrows or get relief from negative feelings’), and (e) Negative consequences (e.g., ‘I neglect important things because of my internet use’). Items were rated on a 5-point scale ranging from 0 (never) to 4 (very often), and higher scores indicate greater compulsive use. We administered the Moroccan Arabic version (Imrani & Touri, 2025b). In the present sample, the CIUS showed good internal consistency (Cronbach’s α = 0.84), indicating satisfactory reliability.

Sociodemographic Information. A brief questionnaire solicited participants’ age, sex, parental occupations, and number of siblings.

Primary Internet Activity and Device Preference. Students indicated their main online activity (e.g., video games, social media, streaming) and preferred device (smartphone, tablet, or computer) to characterize usage patterns.

Screen Time Tracking. Participants provided subjective and objective estimates of their screen time over the previous seven days. The subjective estimates were based on a single retrospective self-report of the total hours spent using smartphones, tablets, and computers during the past week. Objective estimates were obtained from the devices' internal usage logs. During a classroom session, students were guided through a live demonstration showing how to access the “Last 7 Days” screen time report on their smartphones. They learned how to record their total daily screen time and the amount of time spent on each app for each of the seven days. The global subjective weekly estimate was used descriptively, while analyses of temporal patterns relied on day-level objective values (latent class analysis, growth modeling, and mixed-effects models). Telephone measurements were obtained the week prior to the collection of self-reported data. The selection of weeks for the study was meticulous, with the objective of ensuring that the connection times remained consistent throughout the study period. This was achieved by choosing weeks that were not concurrent with school vacations or examination periods, as these periods are known to experience fluctuations in connection times. The research team instructed participants on how to access the built-in screen time statistics on their personal smartphones, such as total screen time over the past seven days and time spent on each application. Participants then manually reported these values on the data sheet. Although these values originate from the devices' internal tracking systems, they are self-reported rather than automatically recorded. For this reason, the data should be considered self-reported rather than fully objective. These data were entered on a summary form to ensure accuracy.

### 3.1 Procedure

The students were informed that the study concerned the everyday digital habits and well-being of adolescents. They were told that they would complete a questionnaire about their screen time, online activities, and general experiences. However, they were not informed in advance that they would be asked to report their device-logged screen time. This was done intentionally to minimize reactivity, or the risk that participants would modify their screen use because they expected the logged values to be checked on a specific date. Once students were in the classroom and had begun the survey, the instructions required them to access the usage statistics on their phones and transcribe the “Last 7 Days” data. Since the device-based information automatically covered the preceding week, participants' behavior during that period could not have been influenced by anticipation of this request.

Data collection then occurred during regular class periods in three phases. First, researchers presented the study aims and procedures and obtained informed consent. Next, students completed the CIUS and sociodemographic and screen‐time questionnaires in groups, with research assistants available to clarify items. Finally, participants extracted and recorded their smartphone usage data under supervision. All data were anonymized prior to analysis to protect confidentiality.

### 3.2 Data analysis

All analyses were performed in R (v4.2.2; R Core Team, 2023). Continuous predictors were standardized, and age was mean-centered. Two latent class analyses were used to identify subgroups based on time spent per week and daily use of specific media. Models with two to seven classes were estimated using the GLCA (generalized latent class analysis) package, and models with two classes were retained (see Vermunt and Magidson, 2002, Nylund et al., 2007, for model specification and class enumeration criteria). Class membership was predicted using multinomial regression, with age, sex, and CIUS scores as covariates. We used a combination of person-centered and time-sensitive statistical techniques to capture variability in screen use throughout the week. We used latent class analysis (LCA) to identify distinct subgroups of adolescents with similar daily usage trajectories. We then modeled daily trajectories using the Richards nonlinear growth curve because it accommodates asymmetric inflection points and plateau–peak–plateau patterns without overfitting (Bates and Watts, 1988, Ritz and Streibig, 2008). Finally, we employed linear mixed-effects models to estimate the association between individual and contextual predictors (e.g., age, sex, and CIU) and daily screen-time fluctuations. Model fit was evaluated using CFI, RMSEA, and χ2. Growth parameters were regressed on CIUS, app usage, age, sex, and school type. Predictors of media-specific time use were examined using LMMs and the lme4 package (Bates et al., 2015). Fixed effects included CIUS, app usage, age, sex, and media category. All mixed-effects models included random intercepts for participants and for schools, in order to account for clustering across the three school contexts (two urban, one rural). Model convergence used the “bobyqa” optimizer, and significance was tested via Satterthwaite's approximation (lmerTest; Kuznetsova et al., 2017). Model fit was assessed with marginal and conditional R2 (Nakagawa & Schielzeth, 2013). For the structural models, we followed the conventional guidelines for interpreting fit indices. Values of 0.95 or higher for the comparative fit index (CFI) and values of 0.06 or lower for the root mean square error of approximation (RMSEA) were considered to indicate a good fit. Values of 0.90 or higher for the CFI and values of 0.08 or lower for the RMSEA were considered to indicate an acceptable fit (Hu & Bentler, 1999). For latent class and nonlinear growth models, we selected the most parsimonious, well-separated solution based on lower values of the Akaike Information Criterion (AIC) and Bayesian Information Criterion (BIC) and higher entropy (Nylund, Asparouhov, & Muthén, 2007). For linear mixed-effects models, we interpreted marginal R^2^ as the variance explained by fixed effects alone and conditional R^2^ as the variance explained by the full model, including random effects (Nakagawa & Schielzeth, 2013). Throughout the manuscript, the abbreviation ps is used to denote p values associated with statistical tests. Statistical significance was evaluated using conventional thresholds (p < 0.05, p < 0.01, and p < 0.001). The intra-class correlation coefficient (ICC) was computed to quantify the proportion of variance attributable to each random-effect level (participants and schools). ICC values close to zero indicate negligible clustering effects, whereas higher values indicate a greater proportion of variance explained by the corresponding grouping factor. The data used in the present study have not been used in any previous publications and are reported here for the first time.

## 4 Results

### 4.1 Descriptive statistics

**Fig. 1:**
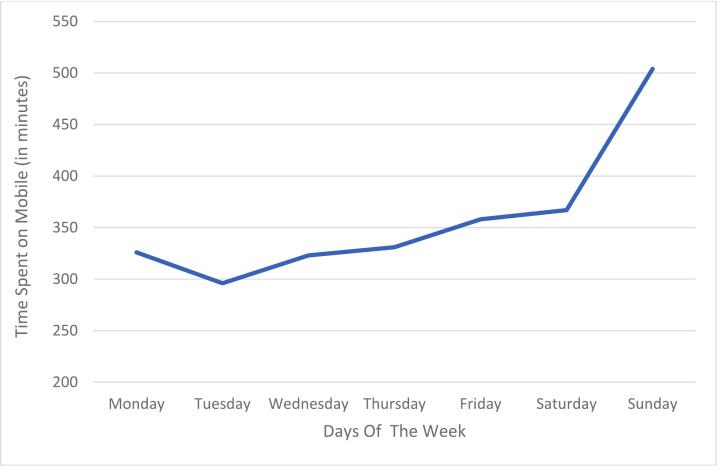
**Weekly trend in adolescents’ daily screen time across seven consecutive days.***Note*. Weekly evolution of mobile screen time, showing a relatively stable usage pattern during weekdays, followed by a significant increase over the weekend.

**Fig. 2:**
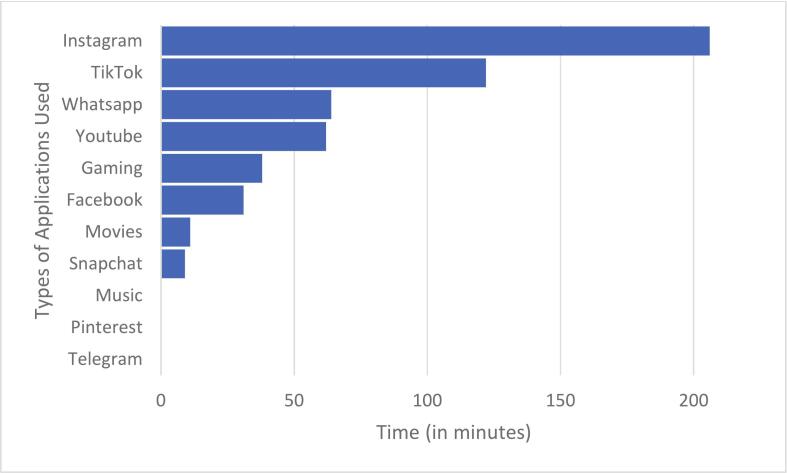
**Average Time Spent on Mobile Applications.***Note*. Average time spent on various mobile applications among students, highlighting significant differences in usage patterns. Instagram emerges as the most time-consuming application, with an average usage of 3 h and 26 min per day, followed by TikTok with 2 h and 2 min, indicating a strong preference for visually engaging and interactive content. Messaging apps such as WhatsApp (1 h and 4 min) and YouTube (1 h and 2 min) also record considerable usage, reflecting a mix of social interaction and media consumption. Gaming (38 min), Facebook (31 min), and Movies (11 min) account for moderate usage, while Snapchat (9 min), Music, Pinterest, and Telegram remain marginally used.

Mean screen time increased across the week and was highest on Sunday, as reflected in the descriptive values reported in Table 1 and in Fig. 1. These day-to-day differences are further confirmed in the mixed-effects model reported below. On weekdays, males reported more screen time than females (e.g., on Wednesday, 323 vs. 282 min), but this gap narrowed on weekends. Platform use also differed by sex: females spent more time on Instagram, TikTok, and WhatsApp, whereas males spent more time on YouTube and gaming applications (Fig. 2). Pinterest and music streaming apps were rarely used in either group. CIUS scores were higher among females (M = 24.91, SD = 11.77) than males (M = 21.05, SD = 10.59). Daily app usage was similar for males (approximately 432 min per day) and females (approximately 416 min per day). Across the week, mean daily screen time ranged from about 4 h and 40 min on weekdays to more than 8 h and 20 min on Sunday (Table 1).

**Table 1:** Full descriptive statistics by sex.

**Variable**	**Category**	**Male *n* (%) or *M* (*SD*)**	**Male median**	**Female *n* (%) or *M* (*SD*)**	**Female median**
Age (years)	—	33.34 (10.14)	22	37.50 (11.16)	24
	Preferred device^a^	Desktop computer	17 (5.1 %)	—	3 (0.9 %)
	Laptop	23 (6.9 %)	—	14 (4.2 %)	—
	Smartphone	118 (35.3 %)	—	154 (46.1 %)	—
	Tablet	1 (0.3 %)	—	2 (0.6 %)	—
	Gaming console	2 (0.6 %)	—	0 (0.0 %)	—
Main internet activity^b^	Social media	103 (30.8 %)	—	134 (40.1 %)	—
	Messaging	3 (0.9 %)	—	8 (2.4 %)	—
	Online gaming	32 (9.6 %)	—	4 (1.2 %)	—
	Information search (non-academic)	6 (1.8 %)	—	5 (1.5 %)	—
	Online shopping/exchanges	1 (0.3 %)	—	0 (0.0 %)	—
	Downloads	5 (1.5 %)	—	10 (3.0 %)	—
	Study-related search	7 (2.1 %)	—	12 (3.6 %)	—
	Gambling/bets	1 (0.3 %)	—	0 (0.0 %)	—
	Other	3 (0.9 %)	—	0 (0.0 %)	—
CIUS score	—	161; 33.34 (10.14)	32.00	173; 37.50 (11.16)	36.00
Screen time (min/day)	Monday	77; 354.38 (153.02)	352.00	111; 305.34 (143.85)	300.00
	Tuesday	83; 309.08 (179.73)	286.00	118; 250.23 (142.87)	251.50
	Wednesday	83; 321.64 (173.91)	324.00	118; 284.77 (172.44)	280.00
	Thursday	83; 314.13 (164.74)	317.00	118; 271.59 (186.27)	243.50
	Friday	83; 350.58 (220.40)	361.00	118; 314.03 (176.35)	310.00
	Saturday	83; 353.33 (218.89)	358.00	118; 328.00 (211.39)	316.50
	Sunday	83; 415.92 (245.36)	420.00	118; 403.31 (219.58)	372.50
App-specific time (min/day)	Facebook	83; 28.31 (80.68)	0.00	117; 28.13 (94.66)	0.00
	YouTube	83; 80.63 (131.74)	30.00	116; 33.45 (53.64)	0.00
	Instagram	83; 151.01 (151.71)	127.00	116; 180.67 (168.84)	135.50
	TikTok	83; 79.66 (204.03)	0.00	116; 95.09 (189.23)	34.00
	WhatsApp	83; 49.28 (84.06)	23.00	116; 64.88 (108.71)	30.50
	Snapchat	83; 6.45 (28.61)	0.00	116; 10.73 (47.36)	0.00
	Telegram	83; 0.24 (2.20)	0.00	116; 0.88 (9.47)	0.00
	Pinterest	83; 0.00 (0.00)	0.00	116; 0.22 (1.67)	0.00
	Music	83; 0.00 (0.00)	0.00	116; 1.51 (11.67)	0.00
	Movies	83; 6.12 (41.21)	0.00	116; 13.76 (72.09)	0.00
	Gaming	83; 63.95 (139.81)	0.00	116; 15.25 (83.85)	0.00

*Note.* CIUS = Compulsive Internet Use Scale; SD = standard deviation; min = minutes. ^a^Preferred device coded as 1 = desktop computer, 2 = laptop, 3 = smartphone, 4 = tablet, 5 = gaming console. ^b^Main internet activity coded as 1 = social media, 2 = messaging, 3 = online gaming, 4 = information search, 5 = online shopping/exchanges, 6 = downloads, 7 = study-related search, 8 = gambling/bets, 10 = other.

### 4.2 Latent class analysis of weekly screen time

Latent class analysis of daily screen time supported a two-class solution. One class (34.7 %) showed relatively low and stable use across the week, whereas the other class (65.3 %) showed higher use with a marked weekend peak. As summarized in Table 2, higher CIUS scores were associated with substantially greater odds of belonging to the high-weekend-peak class rather than the low-stable class (odds ratio [OR] = 2.04). Older age (OR = 1.18) and female sex (OR = 1.40) also predicted membership in the high-use class. Model fit indices for the two-class solution (entropy, log-likelihood, AIC, BIC) are reported in Table 2.

**Table 2:** Summary of model-based associations between CIU, sociodemographic factors, and screen-time outcomes.

**Panel A. Latent class analyses**			
**Outcome/Model**	**Statistic/Predictor**	**Estimate (OR)**	**Notes**
Weekly screen time – class membership (high-weekend-peak vs. low-stable)	Class proportion	High-use: 65.3 %; Low-stable: 34.7 %	Two-class solution; entropy = 1.00; log-likelihood = −6,886.96; AIC = 18,025.91; BIC = 26,128.40
	CIUS score	2.04	Higher CIUS associated with higher odds of high-use membership
	Age (years)	1.18	Older adolescents more likely to be in high-use class
	Sex (female vs. male)	1.40	Females more likely to be in high-use class
Media-specific time – class membership (nonuse vs. active multi-platform)	Class proportion	Nonuse: 45.21 %; Active: 54.79 %	Two-class solution; entropy = 1.00; log-likelihood = −3,929.87; AIC = 9,855.73; BIC = 13,659.25
	CIUS score	0.57	Higher CIUS reduces odds of being in very low/near-nonuse class
	Age (years)	0.87	Younger adolescents more likely to be in active multi-platform class
	Sex (female vs. male)	0.64	Females more likely to be in active multi-platform class

**Panel B. Nonlinear growth models (Richards curves)**			

**Outcome**	**Statistic**	**Value**	**Interpretation**
Weekly total screen time	*R*2	.48	Moderate proportion of variance explained
	F(3, 3)	0.94, *p* = .52	Overall model not statistically significant
	Inflection point	T_1_ = 7.00 (Sunday)	Maximum acceleration just before Sunday (=364.10 min), usage approaching =995 min by week’s end (A = 995.06; d = 0.99; K = 3.90)
Media-specific daily time	*R*2	.34	Moderate proportion of variance explained
	F(3, 7)	1.19, *p* = .382	Overall model not statistically significant
	Inflection point	T_i_ = 4.32 (= Day 4.31)	Acceleration starting around Day 3.78, plateau near 79.86 min (A = 79.86; d = 3.00; K = 8.82)

**Panel C. Linear mixed-effects models**			
**Outcome/Predictor**	**Estimate (minutes)**	**95 % CI**	**Test statistic/Effect size**

**Total daily screen time**	Model: *n* obs. = 13,940; IDs = 201; schools = 3; log-likelihood = −85,681.60; AIC = 171,415.19; BIC = 171,611.30; *R*^2^_marg = .18; *R*^2^_cond = .69		
Age (years)	+16.72	[−2.74, 36.18]	F(1, 183.81) = 2.89, *p* = .091
Sex (female vs. male)	−61.24	[−107.21, −15.26]	F(1, 183.79) = 6.82, *p* = .010
CIUS score (per point)	+3.96	[2.06, 5.86]	F(1, 183.85) = 16.64, *p* < .001
Day of the week (overall)	—	—	F(6, 13,733.25) = 330.75, *p* < .001
Sunday vs. Friday	+79.39	—	Post hoc: *p* < .001
Sunday vs. Saturday	+70.05	—	Post hoc: *p* < .001
Friday/Saturday vs. Monday–Thursday	—	—	All post hoc comparisons: *p* < .001
Primary internet activity (overall)	—	—	F(6, 183.84) = 2.17, *p* = .048
Non-academic information search vs. social media	−180.52	[−316.99, −44.04]	*p* = .010
Study-related search vs. social media	−138.70	[−274.84, −2.56]	*p* = .047
**Media-specific daily minutes**	Model: main predictors only		
CIUS score (per point)	+0.49	[−0.01, 0.99]	F(1, 180.36) = 3.75, *p* = .054
Age, sex, day, siblings, device, activity	—	—	All fixed effects nonsignificant

*Note.* CIUS = Compulsive Internet Use Scale; OR = odds ratio; AIC = Akaike Information Criterion; BIC = Bayesian Information Criterion; *R*^2^_marg = marginal *R*^2^ (fixed effects only); *R*2_cond_ = conditional *R*^2^ (fixed + random effects). Estimates for categorical contrasts are reported relative to the reference categories described in the main text.

### 4.3 Growth modeling of weekly screen time

Nonlinear growth modeling using the Richards curve indicated a progressive increase in daily screen time over the week, with an inflection point at the end of the week and maximum usage reached on Sunday. The model explained approximately 48 % of the variance in the weekly trajectory (R^2^ = 0.48), but the overall test of the sigmoidal curve did not reach statistical significance, F(3, 3) = 0.94, p = 0.52 (Table 2). Descriptively, screen time accelerated toward the end of the week, approaching nearly 1000 min on Sunday, but the specific parametric form of the trajectory should be interpreted with caution.

### 4.4 Linear mixed-effects model on total daily screen time

The linear mixed-effects model on total daily screen time included age, sex, CIUS score, day of the week, number of siblings, parents’ work status, preferred device, and primary internet activity as fixed effects, with random intercepts for participants and schools. Overall model fit and full parameter estimates are presented in Table 2. Sex and CIUS scores emerged as significant predictors of total daily screen time. On average, females reported about 61 min less daily screen time than males (95 % confidence interval [CI] [−107.21, −15.26], p = 0.010). Each additional point on the CIUS was associated with an increase of approximately 4 min of daily screen time (95 % CI [2.06, 5.86], p < 0.001). Age showed a positive but nonsignificant trend, corresponding to roughly 17 additional minutes per year, F(1, 183.81) = 2.89, p = 0.091. Day of the week had a large effect. Sunday displayed the highest levels of use, exceeding both Friday and Saturday by about 70–80 min (all ps < 0.001), and both weekend days showed substantially higher use than Monday through Thursday (all ps < 0.001). Primary internet activity also differentiated total screen time. Compared with adolescents whose main activity was social media, those who mainly engaged in non-academic information search reported approximately 181 fewer minutes per day, and those whose primary activity was study-related search reported about 139 fewer minutes per day (both ps ≤ 0.047). Other predictors, including number of siblings, parents’ work status, and preferred device, did not show reliable effects. The model accounted for about 18 % of the variance in daily screen time at the fixed-effects level and about 69 % when random effects were included (Table 2). Random intercepts were specified for both participants and schools. The school-level random effect was negligible for total daily screen time (SD = 1 min; ICC < 0.001), while the participant-level random effect was substantial (SD = 143 min). For media-specific daily minutes, school-level variation remained negligible (SD = 9 min; ICC = 0.01). Sex and CIUS scores emerged as significant predictors of total daily screen time. Female sex was associated with lower daily screen time compared with males (B =  − 61.24 min, 95 % CI [−107.21, −15.26], p = 0.010). In addition, higher compulsive internet use was associated with greater daily screen time, such that each one-point increase on the CIUS corresponded to an average increase of 3.96 min per day (B = 3.96, 95 % CI [2.06, 5.86], p < 0.001).

### 4.5 Analyses of time spent per media

#### 4.5.1 Latent class analysis of media-specific time

Latent class analysis of minutes spent per media also supported a two-class solution. One class (45.21 %) was characterized by very low or near-nonuse of most platforms, whereas the other class (54.79 %) showed active engagement across multiple platforms. As shown in Table 2, higher CIUS scores were associated with lower odds of belonging to the very low/near-nonuse class (OR = 0.57), indicating that adolescents with more CIU were more likely to exhibit active multi-platform use. Younger age (OR = 0.87) and female sex (OR = 0.64) likewise predicted membership in the active multi-platform class. Fit indices for the media-specific latent class model are reported in Table 2.

#### 4.5.2 Growth modeling of media-specific time

Richards curve modeling of media-specific daily time suggested an increasing trajectory with an inflection point around the middle of the week and a plateau close to 80 min per day. The model explained about 34 % of the variance (R^2^ = 0.34), but the overall test of the curve was not significant, F(3, 7) = 1.19, p = 0.382 (Table 2). These findings suggest a modest tendency for media-specific time to rise and level off mid-week, but again the precise parametric shape should be interpreted cautiously.

#### 4.5.3 Linear mixed-effects model on media minutes

The linear mixed-effects model predicting daily minutes spent on specific media did not yield significant effects for age, sex, day of the week, number of siblings, preferred device, or primary internet activity (Table 2). CIUS scores showed a marginal positive association with media-specific time, such that each additional CIUS point was associated with an increase of roughly half a minute per day, F(1, 180.36) = 3.75, p = 0.054, 95 % CI [−0.01, 0.99]. Overall, CIU showed a small but consistent tendency to predict heavier engagement at the level of individual media, whereas sociodemographic factors played a limited role.

## 5 Discussion

The present study examined adolescent screen time patterns in Morocco using a person-centered latent class approach alongside growth modeling to capture temporal variability in daily use. A seven-day period was selected for the identification of two distinct classes. The first class was characterized by lower and steady use, while the second class was marked by higher use during weekends. Higher scores on the Compulsive Internet Use Scale (CIUS) were strongly associated with membership in the weekend-high class. In addition, older age and female sex further increased the likelihood of belonging to this high-use trajectory. These associations are consistent with theoretical models such as the I-PACE framework (Brand et al., 2016), which posit that reduced executive control and heightened reward sensitivity during adolescence facilitate compulsive digital engagement. These results align with cross-cultural findings indicating that older adolescents and individuals with higher compulsive-use scores are more susceptible to high-intensity patterns (Meerkerk et al., 2009, Van Deursen et al., 2015, Chang, 2025). The clear weekend peak observed in the high-use class aligns with European and Asian data documenting increased leisure time, reduced parental oversight, and greater peer interaction opportunities on non-school days (Rideout, 2016, Verduyn et al., 2017). The present findings extend this pattern to the Moroccan context, where school schedules and family structures deviate from Western norms. The observed age gradient in screen time is consistent with developmental research indicating that increased autonomy in mid- to late adolescence facilitates discretionary media use (Steinberg, 2014).

The gender differences observed in the study, with boys reporting a higher overall screen time, were primarily attributed to their engagement in gaming activities. This observed gender split—boys spending more time gaming and girls using more social/communication platforms—reflects established global trends (Lenhart et al., 2015) and has been replicated in recent multinational surveys of digital engagement (Shannon et al., 2022). Although males reported higher screen time on weekdays, females consistently scored higher on CIU and were more likely to belong to the weekend-high engagement class, reflecting different behavioral pathways to intensive digital use (see Lenhart et al., 2015).

## 6 Methodological considerations, limitations, and future directions

This study builds upon previous research by integrating LCA and LGM, thereby expanding the scope beyond mere aggregate screen time averages to capture the nuances of daily engagement and its predictors. This approach unveils subgroups whose risk for PIU may be underestimated in studies that rely exclusively on weekly averages. Such methods are particularly valuable for public health monitoring, enabling earlier detection of usage escalation patterns. Given the growing evidence that *type* and *timing* of screen activity may be more predictive of psychosocial outcomes than total duration (Chang, 2025), person-centered temporal models represent an important advance.

However, there are several limitations that warrant consideration. First, the data on screen time were self-reported, which can be subject to recall bias and self-presentation effects (Ryding & Kuss, 2020). Subsequent studies should integrate device-logged metrics to enhance the accuracy of the findings. Second, the non-probability sampling strategy imposes limitations on the generalizability of the findings beyond the confines of the studied schools. Third, the cross-sectional design of our study precluded causal inferences regarding the directionality between compulsive use tendencies and screen patterns. The incorporation of developmental, family, and contextual moderators into longitudinal studies would serve to elucidate the underlying causal pathways. Fourth, despite the fact that students obtained the values of their screen time from the tracking tools integrated into their smartphones, the data are self-reported because the values were manually transcribed. This process reduces, but does not eliminate, recall bias or self-presentation effects. Future studies should incorporate direct, automated logging of behavioral data to strengthen measurement accuracy.

Eventually, although the present study was theoretically informed by the I-PACE framework, it focused specifically on compulsive internet use as an observable behavioral manifestation of dysregulated engagement. Other components of the model, such as impulsivity, affective states, and situational triggers, were not directly assessed. Future research could extend this work by integrating intensive daily designs with psychological and contextual measures to examine how these factors interact with CIU to shape short-term fluctuations in digital behavior.

## 7 Implications and conclusion

The weekend surge in screen time seen in this study is crucial for interventions. Behavioral changes like reduced academics, increased socializing, and parental leniency could be driving this. According to brain-development models, adolescents are particularly susceptible to social media and gaming's reinforcing design due to heighetened reward sensitivity and hampered self-regulatory capacities (Steinberg, 2014, Brand et al., 2016), like curated content and intermittent rewards, which may become more important during less constrained times.

Cross-cultural evidence indicates that such temporal patterns are not unique to Morocco. For instance, diary studies in European contexts (Rideout, 2016, Verduyn et al., 2017) and large-scale surveys in East Asia (Kim et al., 2023) have also reported weekend spikes in digital engagement, though there is variation in platform preferences and content type. In MENA countries, where mobile-first internet adoption is prevalent and public leisure infrastructure for adolescents is often limited, weekends may represent a disproportionate time for high-intensity online activity (Bouazza et al., 2023). These patterns likely find their roots in Morocco's digital landscape, which is defined by a high rate of smartphone ownership, reasonably priced data, and a predominantly mobile-first approach to technology use. Morocco is different from many Western countries in that it does not have many educational programs about digital literacy. This could possibly lead to a higher risk of CIU among certain groups in the population. The weekend peak found in this study is important because earlier studies show that a lot of screen time during the weekend can cause sleep problems, bad moods, and stronger emotional reactions (Soriano-Molina et al., 2025, Pazdur et al., 2025). The findings of this study indicate that interventions should target not only total screen exposure but also temporal peaks—particularly on weekends—when usage intensifies.

Therefore, preventive strategies should combine general principles, such as promoting self-regulation skills and parental mediation, with culturally tailored approaches. In Morocco, for instance, this could entail integrating digital literacy into school curricula that explicitly address weekend usage patterns. It could also entail fostering extracurricular activities that facilitate offline socialization during periods of high risk and collaborating with telecom providers to promote healthy usage campaigns. At the policy level, insights can be gained from European initiatives that regulate persuasive design elements in children's applications (European Commission, 2022), as well as from East Asian “cooling-off” interventions that restrict minors' late-night gaming access (Zhao et al., 2024). Such measures may help mitigate the escalation from high weekend use to CIU. They should be adapted to local norms and infrastructure.

The findings of our study underscore the importance of culturally contextualized, time-sensitive assessments of adolescent screen engagement. Interventions should prioritize high-risk subgroups, particularly older adolescents with high CIUS scores, and target peak-use periods to prevent the escalation of PIU. At the policy level, integrating digital literacy education into Moroccan school curricula and promoting parental mediation strategies may serve as effective preventive measures. Given mobile connectivity's role in how young people socialize and learn, it is essential to identify risky profiles to protect the well-being of adolescents.

## Ethics approval statement

All procedures involving human participants performed in this study were in accordance with the Ethics Committee of Hassan II University of Casablanca and the 1964 Helsinki Declaration and its later amendments or comparable ethical standards.

## Patient consent statement

Informed consent was obtained from all participants included in the study.

## Permission to reproduce material from other sources

Not applicable.

## Clinical trial registration

Not applicable.

## Funding statement

This study did not receive any external funding.

## CRediT authorship contribution statement

**Samiha Imrani:** Writing – review & editing, Writing – original draft, Validation, Project administration, Methodology, Investigation, Formal analysis, Data curation, Conceptualization. **Bouzekri Touri:** Writing – review & editing, Writing – original draft, Validation, Supervision, Resources, Project administration, Methodology, Investigation, Funding acquisition, Conceptualization. **Lucia Romo:** Writing – review & editing. **Oulmann Zerhouni:** Writing – review & editing, Validation, Software, Formal analysis, Data curation.

## Declaration of competing interest

The authors declare that they have no known competing financial interests or personal relationships that could have appeared to influence the work reported in this paper.

## Data Availability

The data that support the findings of this study are available at the Open Science Framework: https://osf.io/f4rj2/.
